# Circulating cell-free nucleic acids as potential biomarkers for sarcopenia: a step toward personalized medicine

**DOI:** 10.1186/s40200-017-0299-1

**Published:** 2017-04-20

**Authors:** Gita Shafiee, Ramin Heshmat, Bagher Larijani

**Affiliations:** 1 Chronic Diseases Research Center, Endocrinology and Metabolism Population Sciences Institute, Tehran University of Medical Sciences, Dr shariati hospital, north karegar st, Tehran, 14114 Iran; 2 Endocrinology & Metabolism Research Center, Endocrinology and Metabolism Clinical Sciences Institute, Tehran University of Medical Sciences, Tehran, Iran

**Keywords:** Sarcopenia, Cell-free nucleic acids, Biomarker

## Abstract

Sarcopenia is an age-related loss of muscle mass and function, leading to disability, morbidity and increased mortality in older people. Given the relatively high prevalence and related- outcome of the disease, correct diagnosis, screening, monitoring and treatment of sarcopenia are needed in clinical practice. Recent researches have focused on cell-free nucleic acids, which are released into the circulation following cell death, as a potential biomarker of aging and systematic inflammation. It seems that the diagnosis and treatment of sarcopenia can be possible by the help of the analysis of cell-free nucleic acids as noninvasive method.

## Background

Sarcopenia, a multifactorial geriatric syndrome, is characterized by age-related decline in muscle mass and function [1]. There are multiple intrinsic (biological changes, inflammatory states; etc.) and extrinsic (decreased activity, malnutrition; etc.) factors that participate in the development of sarcopenia [2]. Sarcopenia is a major risk factor of falling, functional limitation, disability, and mortality in the elderly [3].

Given the relatively high prevalence and related- outcome of the disease, correct diagnosis, screening, monitoring and treatment of sarcopenia are needed in clinical practice as well as for the conductance of beneficial interventions. In this regard, the global quantitative data from both genetic, biomarkers and body composition could provide a standardized and international comparable readout for successful care. Moreover, while a number of risk factors and diagnostic methodologies are available, it would be very useful to be able to develop additional predictive tools and risk indexes for this disease. Although, several biological markers have been found to be associated with age-related skeletal muscle decline, but they are not specific to muscle mass and function (inflammatory markers, anabolic hormones, clinical parameters, etc.) [4–7] and many have weakly associated with clinically relevant outcomes. Therefore, a good biomarker for sarcopenia must be specifically for muscle changes, accessible, reliable, non-invasive and cost effective. The ideal biomarker needs to help the clinician to manage disease, select therapy, monitor progression of disease and treatment response [8]. The biomarker could show specific biological processes of sarcopenia to understand specific therapy as a step toward personalized medicine [9]. Personalized medicine could help to distinguish between health and disease and to understand between sarcopenic patients with adverse clinical outcomes that require therapeutic interventions and those that do not.

## Main text

Recent advances in technologies provide an extraordinary capacity to characterize the genetic alterations and pathways in diseases comprehensively and make it possible to develop therapies, prevention and screening based on the genetic makeup of each disease [10].

Circulating cell- free nucleic acids (ccfNA) in various body fluids have been explored as a novel biomarker in a variety of clinical conditions. The first studies concerning the detection of circulating cell free DNA (cf-DNA) was found in various cancers, metastasis and recurrence of tumor [11]. Both apoptosis and necrosis are as the source of the cf-DNA and so, elevated cf-DNA levels have been observed in other conditions such as cardiovascular diseases, sepsis, and trauma [12, 13]. In the recent years, much attention and effort have been put into studies of other circulating nucleic acids, including circulating RNA, microRNA, mitochondrial DNA, mitochondria RNA than cf-DNA [14, 15]. In addition to assessing the quantities of circulating cf-DNA, qualitative features, such as the cf-DNA methylation level, mutations and fragment size have been offered to be useful diagnostic and prognostic markers in various diseases. However, despite the growing utility of circulating nucleic acids assessment, many aspects regarding the regulation of their levels and alterations in the composition of the total circulating nucleic acids in physiological conditions are currently unknown.

Some studies determined that higher levels of cf-DNAs were associated with systematic inflammation and increased frailty and other muscular & neuronal degenerative diseases [16, 17]. Also, there is a molecular signature of sarcopenia that would be useful as a molecular model [18] for the future analysis. Given that sarcopenia is accompanied with increased inflammation, apoptosis and necrosis mediate skeletal muscle fiber loss in age-related mitochondrial enzymatic abnormalities [19]; it might a good idea that cell free nucleic acids could serve as a candidate biomarker.

## Conclusions

It seems that cell free nucleic acids could be suggested as a potential biomarker to access the proper therapeutic policy of sarcopenia in personalized medicine and also, to distinguish early stage of disease to reduce the progress of sarcopenia and prevent physical disability.

## Abbreviations

ccfNA: Circulating cell- free nucleic acids
cf-DNA: Circulating cell free DNA

## References

[1] Cruz-Jentoft AJ, Baeyens JP, Bauer JM, Boirie Y, Cederholm T, Landi F, Martin FC, Michel JP, Rolland Y, Schneider SM (2010). Sarcopenia: European consensus on definition and diagnosis: report of the European working group on sarcopenia in older people. Age Ageing.

[2] Walston JD (2012). Sarcopenia in older adults. Curr Opin Rheumatol.

[3] Houston DK, Nicklas BJ, Zizza CA (2009). Weighty concerns: the growing prevalence of obesity among older adults. J Am Diet Assoc.

[4] Cesari M, Penninx BW, Pahor M, Lauretani F, Corsi AM, Rhys Williams G, Guralnik JM, Ferrucci L (2004). Inflammatory markers and physical performance in older persons: the InCHIANTI study. J Gerontol A Biol Sci Med Sci.

[5] Cesari M, Penninx BW, Lauretani F, Russo CR, Carter C, Bandinelli S, Atkinson H, Onder G, Pahor M, Ferrucci L (2004). Hemoglobin levels and skeletal muscle: results from the InCHIANTI study. J Gerontol A Biol Sci Med Sci.

[6] Perrini S, Laviola L, Carreira MC, Cignarelli A, Natalicchio A, Giorgino F (2010). The GH/IGF1 axis and signaling pathways in the muscle and bone: mechanisms underlying age-related skeletal muscle wasting and osteoporosis. J Endocrinol.

[7] Morley JE, Baumgartner RN, Roubenoff R, Mayer J, Nair KS (2001). Sarcopenia. J Lab Clin Med.

[8] Morrow DA, de Lemos JA (2007). Benchmarks for the assessment of novel cardiovascular biomarkers. Circulation.

[9] Agyeman AA, Ofori-Asenso R (2015). Perspective: does personalized medicine hold the future for medicine?. J Pharm Bioallied Sci.

[10] Stratton MR (2011). Exploring the genomes of cancer cells: progress and promise. Science.

[11] Yuan H, Zhu ZZ, Lu Y, Liu F, Zhang W, Huang G, Zhu G, Jiang B (2012). A modified extraction method of circulating free DNA for epidermal growth factor receptor mutation analysis. Yonsei Med J.

[12] Ziegler A, Zangemeister-Wittke U, Stahel RA (2002). Circulating DNA: a new diagnostic gold mine?. Cancer Treat Rev.

[13] Butt AN, Swaminathan R (2008). Overview of circulating nucleic acids in plasma/serum. Ann N Y Acad Sci.

[14] Tsang JC, Lo YM (2007). Circulating nucleic acids in plasma/serum. Pathology.

[15] Yu M (2012). Somatic mitochondrial DNA mutations in human cancers. Adv Clin Chem.

[16] Jylhava J, Nevalainen T, Marttila S, Jylha M, Hervonen A, Hurme M (2013). Characterization of the role of distinct plasma cell-free DNA species in age-associated inflammation and frailty. Aging Cell.

[17] Swarup V, Srivastava AK, Padma MV, Rajeswari MR (2011). Quantification of circulating plasma DNA in Friedreich’s ataxia and spinocerebellar ataxia types 2 and 12. DNA Cell Biol.

[18] Giresi PG, Stevenson EJ, Theilhaber J, Koncarevic A, Parkington J, Fielding RA, Kandarian SC (2005). Identification of a molecular signature of sarcopenia. Physiol Genomics.

[19] Cheema N, Herbst A, McKenzie D, Aiken JM (2015). Apoptosis and necrosis mediate skeletal muscle fiber loss in age-induced mitochondrial enzymatic abnormalities. Aging Cell.

